# Approach to hyponatremia in congestive heart failure: a survey of Canadian specialist physicians and trainees

**DOI:** 10.1186/s40697-016-0094-9

**Published:** 2016-01-20

**Authors:** Amanda Miller, Bonnie Kuehl, Karthik Tennankore, Steven Soroka

**Affiliations:** QEII Halifax Sciences Centre, 5092 Dickson Building, 5820 University Avenue, Halifax, Nova Scotia B3H 1V8 Canada; Scientific Insights Consulting Group Inc., 1993 Balsam Ave., Mississauga, Ontario L5J 1L3 Canada

**Keywords:** Hyponatremia, Congestive heart failure, Education

## Abstract

**Background:**

Hyponatremia is a recognized complication of congestive heart failure (CHF) and is associated with reduced survival. Therefore, early identification and appropriate management of hyponatremia is important. The aim of this study was to determine the general approach amongst Canadian healthcare practitioners and trainees to the identification and management of hyponatremia complicating CHF.

**Methods:**

Respondents completed 15 multiple-choice style questions in 3 case scenarios regarding the approach to management of hyponatremia complicating CHF using an online survey on UKidney.com between November 2012 and May 2013. Results were presented as a proportion of averaged correct/incorrect responses amongst Canadian nephrologists, cardiologists, internists and trainees in each of two domains; pathophysiology and management. Management was further subdivided into correct and incorrect use of diuretic therapy, hypertonic saline, oral urea tablets, vasopressin receptor antagonists (vaptans) and rate of sodium correction. Correct responses were determined by an expert panel of Canadian nephrologists and cardiologists based on review of evidence informed guidelines and current recommendations.

**Results:**

There were 1757 responses to our online survey amongst 455 Canadian respondents, 1139 of which were from cardiologists, nephrologists, general internists, or trainees. Overall, the pathophysiology governing hyponatremia in CHF was correctly identified 68.7 % of the time (*n* = 380 responses, averaged over 4 questions). Hyponatremia was managed inappropriately 43.6 % of the time, with trainees scoring best overall with correct responses 60.3 % of the time (*n* = 759 responses, over 11 questions). Importantly, an incorrect rate for sodium correction was selected 61.1 % of the time overall, (*n* = 211 responses, averaged over 3 questions).

**Conclusions:**

This study identified that there are differences in the understanding of pathophysiology and management strategies for hyponatremia in the context of CHF amongst Canadian specialist physicians and trainees. A more consistent approach to hyponatremia is required and might best be achieved through formal knowledge translation.

**Electronic supplementary material:**

The online version of this article (doi:10.1186/s40697-016-0094-9) contains supplementary material, which is available to authorized users.

## Abrégé

**Mise en contexte:**

L’hyponatrémie, une complication inhérente de l’insuffisance cardiaque congestive (ICC), compromet de façon significative la survie des patients affectés. Dès lors, l’identification précoce de ce trouble chez les patients ainsi que leur prise en charge adéquate revêtent une importance cruciale.

**Objectif de l’étude:**

Cette étude visait à définir l’approche préconisée par les professionnels de la santé canadiens et leurs stagiaires en regard de l’identification et de la prise en charge des cas d’hyponatrémie qui résultent d’une complication de l’ICC.

**Cadre et type d’étude:**

Cette étude descriptive a été réalisée à partir des réponses colligées à la suite d’un sondage électronique envoyé à des médecins spécialistes et médecins résidents canadiens.

**Participants:**

Les participants de cette étude sont un groupe de professionnels de la santé (néphrologues, cardiologues, spécialistes en médecine interne et résidents) canadiens ayant répondu à un sondage en ligne sur UKidney.com, entre novembre 2012 et mai 2013.

**Mesures:**

Les bonnes réponses obtenues lors du sondage de 15 questions relatives à la détection et la prise en charge ont été colligées.

**Méthode:**

Les résultats ont été présentés en proportion des moyennes de bonnes et de mauvaises réponses aux questions parmi les différentes spécialités des participants pour deux domaines précis : la physiopathologie de l’hyponatrémie et la prise en charge du patient. Ce deuxième aspect a par la suite été subdivisé en prise de décision correcte ou incorrecte en regard des interventions suivantes : l’administration d’un traitement diurétique ou d’une solution saline hypertonique, la prescription de comprimés d’urée par voie orale ou d’antagonistes des récepteurs de vasopressine (les « Vaptans »), ou alors une correction du taux de sodium. Les bonnes réponses aux questions avaient été déterminées au préalable, par un comité d’experts (néphrologues et cardiologues canadiens), sur la base d’une révision des données probantes, des lignes directrices et des recommandations actuelles en matière d’intervention auprès des patients souffrant d’hyponatrémie.

**Résultats:**

De tous les professionnels de la santé qui ont répondu au sondage, 455 étaient Canadiens. En répondant à un minimum d’une question du sondage, ces derniers ont fourni un total de 1757 réponses pour analyse. De ce total, 1139 provenaient de néphrologues, de cardiologues, de spécialistes en médecine interne ou de résidents. Dans l’ensemble, les questions traitant de la physiopathologie de l’hyponatrémie conséquente d’une ICC ont été plutôt bien réussies, avec un taux d’identification du trouble à 68,7 % (*n* = 380 bonnes réponses, calculées sur une moyenne de 4 questions). Il en fut autrement de la portion du sondage qui concernait la prise en charge des patients souffrant d’hyponatrémie où le taux de réussite s’est avéré passablement faible (*n* = 759 réponses, calculées sur une moyenne de 11 questions) ; les résidents ayant obtenu la meilleure note avec une moyenne de 60,3 % de bonnes réponses. Un fait étonnant a été noté, soit que 61,1 % des répondants (*n* = 211 bonnes réponses, calculées sur une moyenne de 3 questions) n’aient pu effectuer la correction du taux de sodium adéquatement.

**Limites de l’étude:**

Cette étude est limitée du fait qu’elle s’appuie sur un sondage auquel les participants répondaient sur une base volontaire et parce que de l’exactitude des réponses se dégageait un parti pris inhérent. De plus, l’échantillonnage du sondage s’avère limité.

**Conclusion:**

L’étude a permis de constater la présence de différences notables dans la compréhension de la physiopathologie et dans les stratégies de prise en charge des patients qui souffrent d’hyponatrémie conséquente à l’ICC chez les professionnels de la santé canadiens. La prise en charge de ces patients nécessite une stratégie d’intervention plus cohérente de la part des médecins spécialistes et des résidents, et cet objectif pourrait vraisemblablement être atteint par un meilleur transfert des connaissances.

## What was known before

Hyponatremia is a common electrolyte disorder that complicates approximately 20 % of hospital admissions for congestive heart failure, and its inappropriate assessment and management may have harmful consequences. Currently there are no Canadian guidelines to aid in the management of hyponatremia, which may lead to inconsistencies amongst Canadian healthcare practitioners and trainees in their approach to the work-up and management of this condition.

## What this adds

We found that in a group of Canadian physicians and trainees, there was substantial variability in the understanding of the pathophysiology and subsequent treatment of hyponatremia in congestive heart failure, with trainees demonstrating a better knowledge base overall than their staff counterparts.

## Background

Hyponatremia is defined as a serum sodium of less than 135 meq/L and is the most common electrolyte disorder to occur in both inpatient and outpatient settings [1]. It is most prevalent in elderly populations [2–5] and complicates 15 to 30 % of all hospitalizations, with up to two thirds of recognized cases being hospital-acquired or iatrogenic [6–8]. A low serum sodium is independently associated with an increase in resource consumption and a 60-fold greater mortality rate than in those without documented hyponatremia [9, 10].

Numerous studies have explored the link between hyponatremia and congestive heart failure (CHF) [11–14]. Heart failure is often associated with hyponatremia through complex mechanisms which include hemodynamic, hormonal, and biochemical factors in addition to the effects of diuretic therapy which is often used in the management of heart failure [11]. Data suggests that approximately 20 % of patients hospitalized with heart failure have hyponatremia, which is associated with shorter median survival [12–14].

Some options for sodium correction include diuretic therapy, oral urea tablets, hypertonic saline, and vasopressin receptor antagonists or *vaptans.* Currently however, there are no Canadian guidelines to govern management of this condition and the inappropriate use of any one of these therapies may have harmful consequences. In all cases, correction of serum sodium must be guided by the underlying cause of the hyponatremia and the available evidence for both pharmacological and non-pharmacological approaches. Thus far, there has not been an assessment of the appropriateness of the management of hyponatremia amongst Canadian healthcare practitioners. With this, identified gaps in knowledge could be used to guide further education and knowledge translation to improve outcomes in the management of hyponatremia at a national level.

The aim of this study was to determine the general understanding of hyponatremia in the context of congestive heart failure amongst Canadian healthcare practitioners (nephrologists, cardiologists and internists) and trainees using a case-based survey. We hypothesized that there would be isolated knowledge gaps identified amongst specialists and trainees with variable understanding of the pathophysiology and management of hyponatremia between these groups.

## Methods

### Study design

An online survey was created to investigate the pathophysiology and management strategies for hyponatremia in patients with heart failure. The survey was designed around three patient cases with increasing complexity. These cases all described hypervolemic hyponatremia on the basis of reduced effective circulating volume due to heart failure, heart failure with acute kidney injury, and heart failure with myocarditis and sepsis. A team of three nephrologists and three cardiologists designed the cases, with one nephrologist and cardiologist combination responsible for each case. Cases were then discussed amongst the group of six specialists, and the correct answer for each question was determined based on a review of the available literature. In the infrequent situation where there was disagreement between case writers, the correct answer was chosen by a majority vote. These cases were posted on UKidney.com and were open for Canadian healthcare practitioners to assess and complete. To raise awareness about the survey and to encourage participation by Canadian nephrologists and internal medicine specialists, the survey was highlighted on the UKidney homepage with an invitation sent via email to all registered UKidney members. It was also highlighted on the homepage of the Canadian Cardiovascular Society (CCS) website and advertised in a monthly newsletter sent to all CCS members, publicized in an email to members of the Canadian and Quebec Heart Failure societies, and promoted by the Hyponatremia Working Group and other physicians at their respective health centers and universities. The survey was also posted in the heart failure guidelines section of the CCS website. The answers/expert opinions were posted on UKidney following conclusion of the survey.

Each case was presented as a short vignette detailing a patient with hypervolemic hyponatremia, including relevant background information and additional laboratory values. Four to five potential assessment or treatment options were then offered as multiple-choice questions at various decision points within each case, and respondents were instructed to select the one most correct response. Data was collected from November 26, 2012 to May 20, 2013 and included responses to the questions as well as information on survey participants (specialty, work setting, country of residence). Participants were not required to provide responses to all questions or complete all cases. A complete copy of the survey (case study and questions) is provided in Additional file 1.

### Analysis

Responses from Canadian healthcare practitioners including nephrologists, cardiologists, internists, and trainees (collectively nephrology fellows, cardiology fellows, and GIM residents/fellows) were included in the analysis. Responses to individual questions were counted separately and separated by speciality. Responses were divided into pathophysiology (4 questions) and overall management (11 questions). Management was subcategorized into five areas including rate of sodium correction and the use of diuretics, hypertonic saline, urea tablets, and vaptans. To be included in the analysis of a specific subcategory, a certain treatment option (for example, vaptan use) only need be listed as a possible option (correct or incorrect) for a given question. As each question was composed of 4–5 selection options, several questions were counted in different subcategories, depending on what was provided as possible answer options. Additional file 2 depicts which questions were included in the assessment of the pathophysiology and varying management strategies for hyponatremia in CHF, as well as the number of incorrect responses, or *distractors*, for each multiple-choice question.

The average proportion of correct (or incorrect) responses within each category/subcategory was determined by adding the total number of correct responses in any one question set and dividing by the total number of responses (not respondents) for that question set. This produced a percentage representing the correct (or incorrectly chosen) responses for that group. The results were tabulated in this manner to avoid overrepresentation of certain questions with smaller numbers of responses.

## Results

### Study population

**Fig. 1:**
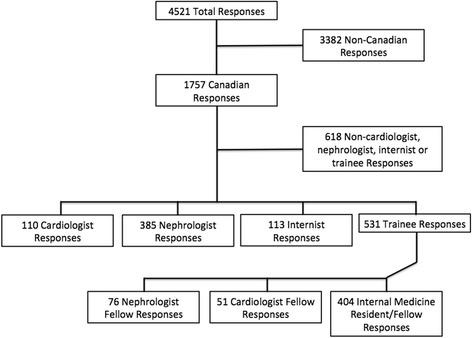
Flow diagram for inclusions/exclusions

Of the 1884 individuals that initiated the survey worldwide (4521 responses), 455 were Canadian respondents who answered at least one question (total of 1757 responses). Most Canadian participants completed the survey early; 57 % of the surveys were started before January 1, 2013, approximately 1 month after the survey opened. Of the three cases posted to the UKidney website, case 1 had the most respondents (*n* = 278 answering any question). The majority (56.7 %) of participants practiced in teaching hospitals and 31.9 % in community hospitals or clinics. Most respondents were nephrologists, cardiologists, or internists (staff + trainees, *n* = 165 individuals, *n* = 1139 responses), with a high proportion of trainees (44 % of respondents). For this reason, to ease interpretation of the results, only these four groups were included in the analysis. See flow diagram below, Fig. 1.

### Pathophysiology

**Fig. 2:**
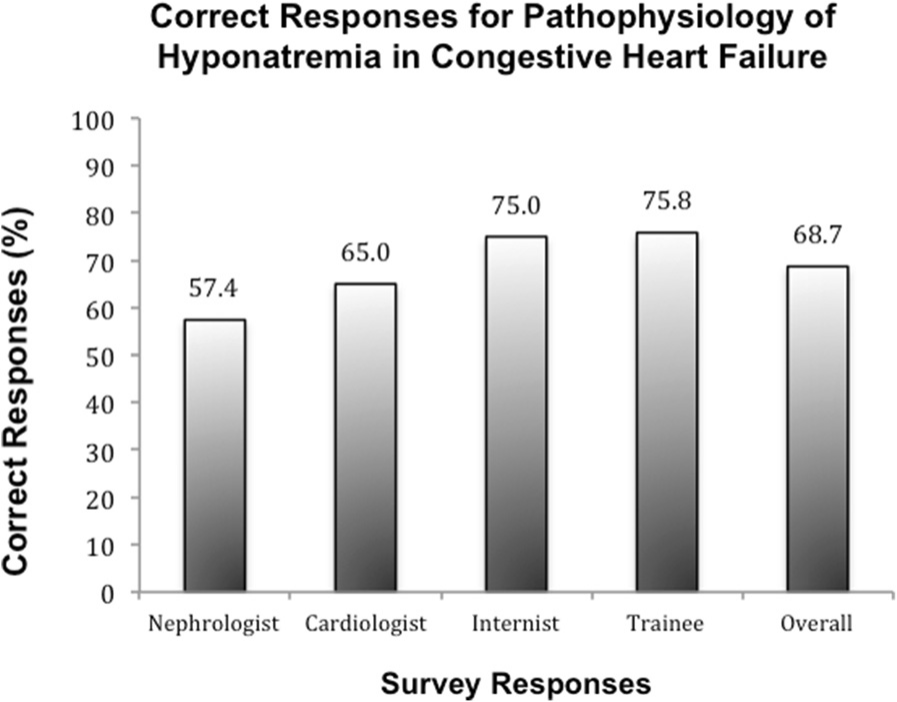
Overall responses for assessment of pathophysiology behind hyponatremia, by practice/clinician subgroup

Four questions from two cases assessed a participant’s ability to identify the underlying pathophysiology behind the development of hyponatremia. Overall, 68.7 % of respondents accurately identified the underlying process leading to development of hyponatremia, Fig. 2. This ranged from a low of 51.2 % of participants identifying the pathophysiology behind hyponatremia associated with kidney dysfunction to 88.9 % correctly identifying the effects of low effective circulating volume on sodium balance.

### Management

**Fig. 3:**
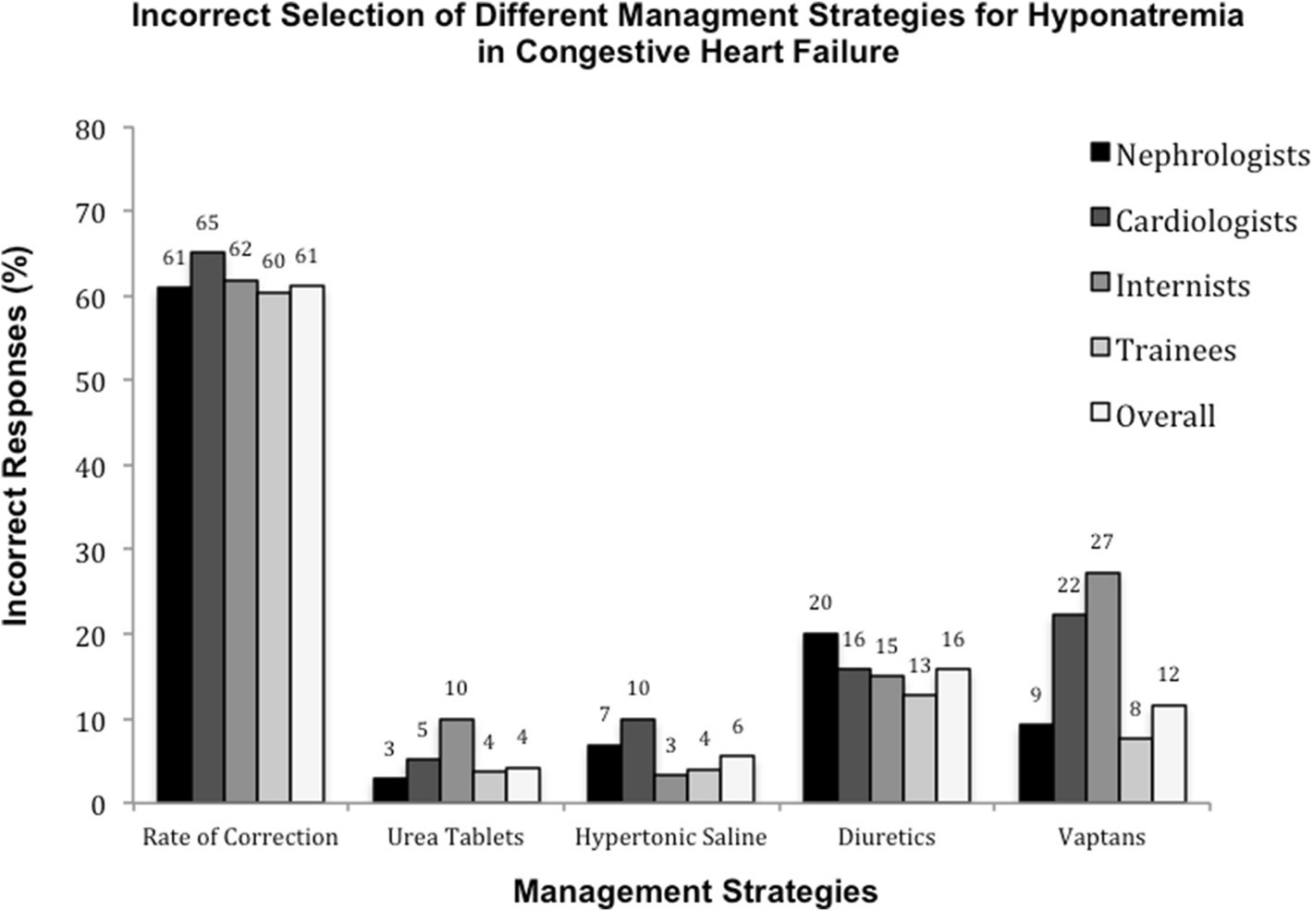
Overall incorrect selection responses for different management strategies, by subgroup

Eleven questions assessed the management of patients with hyponatremia. Overall, the correct response was selected only 56.4 % of the time, based on an equal weighting of the 11 questions addressing management strategies, Fig. 3.

**Fig. 4:**
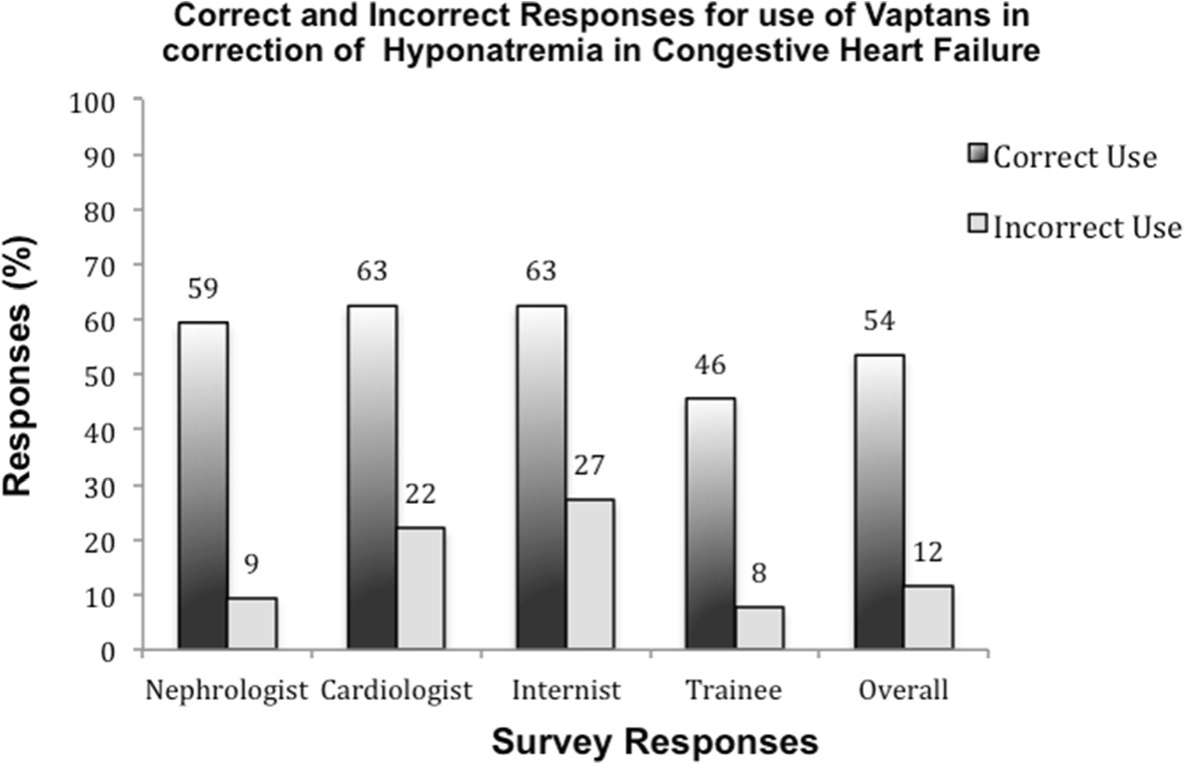
Correct and incorrect responses for use of vaptans in treatment of hyponatremia, by subgroup

Based on the responses to three questions in this survey, participants selected the appropriate rate of sodium correction 38.9 % of the time. The responses to six questions identified that appropriate diuretic use was selected 50 % of the time. Conversely, diuretics were chosen inappropriately 15.8 % of the time. Hypertonic saline was presented as a possible therapy for hyponatremia in five questions. In no question, stem was hypertonic saline considered the correct management. Overall, 94.5 % of respondents recognized a more appropriate therapy for the correction of hyponatremia than hypertonic saline. Oral urea tablets were offered as a potential treatment strategy in five questions. In no scenario, oral urea was the most appropriate management option. Urea tablets were selected by 4.2 % of respondents overall in the management of hyponatremia. Internists were the most likely to opt for using urea tablets for treatment of hyponatremia, choosing this option 10 % of the time overall. There were a total of five questions where participants could choose to use a vaptan for correction of hyponatremia. In two questions, vaptan use was considered the most correct response, whereas the selection of a vaptan was inappropriate in three of the five questions. The participants correctly chose to use a vaptan 53.5 % of the time but failed to identify vaptan use as an appropriate therapy 46.5 % of the time. Conversely, vaptans were chosen inappropriately 11.6 % of the time (from 7.6 to 27.3 %) when a more appropriate answer was presented. Trainees were least likely to use a vaptan (either correctly or incorrectly), Fig. 4.

## Discussion

The aim of this study was to determine the general understanding of hyponatremia in the context of congestive heart failure through a survey of Canadian healthcare practitioners. We identified that Canadian healthcare providers appear to have a less than expected understanding of the pathophysiology and management strategies for hypervolemic hyponatremia. Trainees responded similarly or better than staff specialists in every aspect (pathophysiology and management) and while these findings were not confirmed with statistical analyses due to small sample sizes, this remains a hypothesis-generating observation.

There may be a number of possible explanations for why trainees outscored their staff counterparts in the identification and management of hyponatremia. Firstly, this may reflect the fact that most internal medicine, cardiology, and nephrology trainees are hospital-based and thus would have a reasonable familiarity with the cases presented in the survey based on patients assessed in emergency rooms and on inpatient wards. In this context, trainees may be more frequent users of current literature and therefore more aware of appropriate management strategies for hyponatremia in heart failure. Additionally, this may reflect a relatively standardized education program at the level of a clinical trainee, which further enhances the trainee’s knowledge of hyponatremia.

This study highlighted a number of knowledge gaps in the treatment of hyponatremia amongst Canadian healthcare professionals. One of the cornerstones in the management of hyponatremia is following a strict rate of sodium correction in chronic hyponatremia, but most Canadian participants failed to select the appropriate correction rate. This is concerning as inappropriate targets for sodium correction can have catastrophic consequences. Notwithstanding the low response rates, trends within the specialty groups suggest that cardiologists are more conservative than nephrologists in choosing rates of correction. The cardiologists consistently chose lower rates of sodium correction irrespective of the complexity of the patient, which may be the result of differences in education programs between specialties or variability in familiarity with the management of hyponatremia between the two groups. While the numbers selecting hypertonic saline for the management of stable hypervolemic hyponatremia were small, it is still concerning that this potentially dangerous therapy was chosen at all. Cardiologists and nephrologists were most likely to use hypertonic saline inappropriately, and trainees and general internists least likely to do so. This may be due to the fact that in many centers, the management of hyponatremia has shifted towards the realm of the general internist. In addition, trainees are expected to understand the management of hyponatremia regardless of their chosen specialty. It may be that without interval updates on current management strategies for hyponatremia, staff cardiologists, and nephrologists are more inclined to use outdated therapies than are their colleagues in general internal medicine who consult on this issue regularly. While the tendency to manage stable hyponatremia with hypertonic saline was low overall, it is apparent that Canadian healthcare providers are not in consensus on when to initiate hypertonic saline therapy (a potentially harmful and contraindicated therapy in stable hypervolemic hyponatremia), and this is a concerning fact that must be addressed. Lastly, internists and cardiologists were more inclined to opt for vaptan therapy than were other physicians. This may be a reflection of their level of comfort dealing with acutely ill patients with hyponatremia in the context of decompensated heart failure and renal dysfunction. Nephrologists may be less likely to use vaptans due to an unfamiliarity in this population as they are often consulted only once renal failure is advanced, requiring ultrafiltration to manage extracellular volume overload and concomitant hyponatremia. In addition, the relative unwillingness of trainees to opt for vaptan therapy may reflect either a detailed understanding of the literature (which to date have failed to demonstrate any hard endpoint benefit), or a lack of familiarity with this relatively novel therapy. In this situation, vaptans have not been rigorously studied and the data surrounding their use remains somewhat controversial. For this reason, it is not surprising that responses regarding the correct use of vaptans were varied.

This study would suggest that there is a need for better education surrounding the identification and management of hyponatremia in CHF; however, sample sizes were small and thus, these results may serve only as a suggestion of possible trends amongst healthcare provider subgroups. Collectively, however, these observations would imply that there remains significant uncertainty amongst Canadian healthcare providers about the etiology of hyponatremia in CHF and the management strategies for hypervolemic hyponatremia. This highlights the need not only for a structured approach to hyponatremia at the trainee level but also for ongoing educational updates throughout the span of a physician’s career, perhaps with continuing medical education (CME) credit. This may be the highest yield intervention given the observation that staff physicians scored substantially worse than did trainees who are earlier in their careers and perhaps closer to a formal education curriculum.

This study had a number of strengths, including the fact that the survey cases were designed by a team of cardiologist and nephrologists, as opposed to one person or specialist group in isolation. The cases were promoted to cardiologists, nephrologists, and general internists through various internet associations and local hospitals resources. There were a large number of respondents, and response numbers overall were large, despite small sample sizes in several subcategories.

It is important, however, to recognize that this study had a number of significant limitations. Firstly, data was compiled from the assessment of responses to a voluntary survey, which implies innate sample bias. The survey was posted to the UKidney.com website which is a learning tool for nephrologists and those with an interest in nephrology. As such, those who chose to complete the survey might be systematically different from the general medical community on the basis of being highly motivated with an underlying interest in hyponatremia and a desire to participate in a knowledge assessment survey. Alternatively, respondents may have been those with a relative deficiency in their understanding of hyponatremia and a desire to enhance their knowledge. The number of responses within individual specialities was small, and therefore, differences in responses between specialities are explorative. Furthermore, the use of multiple-choice questions as a survey tool has the potential to mislead or oversimplify responses to complex medical problems, which may modify survey results and add to response bias. Another notable limitation is that what has been determined the correct response to the survey questions was largely based on expert opinion following review by a panel of Canadian nephrologists and cardiologists. This is because in many aspects of hyponatremia management, there are no clear evidence-based guidelines, and expert opinion is all that exists to guide therapy decisions. Lastly, this survey was presented to participants as a series of three cases, each progressively more complex than the previous. There was a high attrition rate from case 1 to case 3 (which progressed in terms of complexity). While this may simply reflect a loss of interest, it also may represent unfamiliarity and a lack of confidence amongst healthcare providers when it comes to more complex cases of hyponatremia. This in itself may result in a certain degree of sample bias given that it is possible that only the most informed and confident respondents persevered through to the more difficult cases.

## Conclusions

We found in a group of Canadian physicians and trainees that there is substantial variability in the understanding of the pathophysiology and subsequent treatment of hyponatremia in congestive heart failure, with trainees demonstrating a better knowledge base overall than their staff counterparts. The optimal treatment pathways are not well defined in patients with CHF and hyponatremia, resulting in widely diverse treatment patterns and a lack of a uniformly accepted standard of care. This fact identifies a need for the development and teaching of a more consistent approach to the management of hyponatremia in these patients. In order to improve the management of hyponatremia, physicians must increase their knowledge base and then apply their new understanding in practice.

## Additional files

Additional file 1:
**The complete survey cases and questions posted to Ukidney.com from November 26, 2012 to May 20, 2013.** This online survey consisted of 15 multiple-choice style questions in 3 case scenarios regarding the approach to and management of hyponatremia complicating CHF. The survey was posted to Ukidney.com and responses were collected between November 2012 and May 2013. (DOCX 34 kb) Additional file 2:
**Individual questions assessing the pathophysiology and management strategies for hyponatremia.** The complete list of questions included in the assessment of the pathophysiology and varying management strategies for hyponatremia are listed. The number of incorrect responses (*distractors*) for each multiple-choice question are included for completeness. (PNG 126 kb)

## References

[1] Gosch M, Joosten-Gstrein B, Heppner HJ, Lechleitner M (2012). Hyponatremia in geriatric inhospital patients: effects on results of a comprehensive geriatric assessment. Gerentology.

[2] Bourdel-Marchasson I, Laksir H, Puget E (2010). Interpreting routine biochemistry in those aged over 65 years: a time for change. Maturitas.

[3] Soiza RL, Cumming K, Clarke JM, Wood KM, Myint PK (2014). Hyponatremia: Special Considerations in Older Patients. J Clin Med..

[4] Kugler JP, Hustead T (2000). Hyponatremia and hypernatremia in the elderly. Am Fam Physician.

[5] Chassagne P, Druesne L, Capet C, Ménard JF, Bercoff E (2006). Clinical presentation of hypernatremia in elderly patients: a case control study. JAGS.

[6] Baran D, Hutchinson TA (1984). The outcome of hyponatremia in a general hospital population. Clin Nephrol.

[7] Hoorn E, Lindemans J, Zietse R (2004). Hyponatremia in hospitalized patients: epidemiology, etiology and symptomatology. J Am Soc Nephrol.

[8] Hawkins RC (2003). Age and gender as risk factors for hyponatremia and hypernatremia. Clin Chim Acta.

[9] Anderson RJ, Chung HM, Kluge R, Schrier RW (1985). Hyponatremia: a prospective analysis of its epidemiology and the pathogenetic role of vasopressin. Ann Intern Med.

[10] Wald R, Jaber BL, Price LL, Upadhyay A, Madias NE (2010). Impact of hospital-associated hyponatremia on selected outcomes. Arch Intern Med.

[11] Triposkiadis F, Starling R, Boudoulas H, Giamouzis G, Butler J (2012). The cardiorenal syndrome in heart failure: cardiac? renal? syndrome?. Heart Fail Rev.

[12] Lee WH, Packer M (1986). Prognostic importance of serum sodium concentration and its modification by converting-enzyme inhibition in patients with severe chronic heart failure. Circulation.

[13] Verbalis JG, Goldsmith SR, Greenberg A, Schrier RW, Sterns RH (2007). Hyponatremia treatment guidelines 2007: expert panel recommendations. Am J Med.

[14] Ghali JK, Tam SW (2010). The critical link of hypervolemia and hyponatremia in heart failure and the potential role of arginine vasopressin antagonists. J Card Fail.

