# Supplementary material for: Approach to hyponatremia in congestive heart failure: a survey of Canadian specialist physicians and trainees
Source: Can J Kidney Health Dis. 2016 Jan 20;3:4. doi: 10.1186/s40697-016-0094-9 (PMC4719575; doi:10.1186/s40697-016-0094-9)
Supplement: Additional file 1: — The complete survey cases and questions posted to Ukidney.com from November 26, 2012 to May 20, 2013. This online survey consisted of 15 multiple-choice style questions in 3 case scenarios regarding the approach to and management of hyponatremia complicating CHF. The survey was posted to Ukidney.com and responses were collected between November 2012 and May 2013. (DOCX 34 kb) [file 40697_2016_94_MOESM1_ESM.docx]

**Cases and Surveys Posted on Ukidney.com from December 12, 2012 to May 20, 2013.**

- Consensus agreement for the correct answer to each question is bolded and highlighted with an asterisk

**Case 1: Basic Case**

**Case Introduction**

- A 70-year-old male with type 2 diabetes, hypertension, two previous myocardial infarctions admitted with progressive symptoms of heart failure over the past two months and weight gain of over 10 kg. His large anterior wall myocardial infarction one year ago required stent deployment in the left anterior descending and right coronary arteries. The left ventricular ejection fraction (LVEF) on last echocardiogram (ECHO), approximately four months ago, was 25%.
- The patient was initially seen in emergency at another hospital and furosemide was increased to 80 mg orally (po) once daily, hydrochlorothiazide (HCTZ) (25 mg po every other day) and ramipril (2.5 mg po twice daily) were added. He was discharged.
- One week later the patient presents in emergency again with further weight gain, increased dyspnea and severe bilateral pitting edema up to his thighs. The patient states he had not been told about fluid restriction and has been drinking water ad libitum.
- On examination his weight is 98 kg, oxygen (O_2_) saturation 85% on room air (RA), blood pressure (BP) 100/50, pulse 96 beats per minute (bpm), jugular venous pressure (JVP) 6 cm above sternal angle (ASA) at 30°, bilateral coarse crackles in the chest, and chest X-ray shows pulmonary edema and pleural effusions. Laboratory values: serum creatinine 150 μM, serum sodium 128mM, serum potassium 3.2mM, B-type natriuretic peptide (BNP) 2300 pg/ml, serum glucose 9.3 mM, and glycated hemoglobin (A1C) 6.8%. Urine was not sent for analysis.

**Basic case Question 1-1:** **What is your interpretation of the laboratory values?**

1. This patient has increased total body water and decreased total body sodium
2. Serum glucose is a significant contributor to the hyponatremia
3. The low serum potassium protects against a further drop in the serum sodium
4. **This patient has increased total body water and decreased sodium concentration in his effective circulating volume that has led to a shift of water into the cells ***

**Basic Case continued:**

- The patient is admitted to the Medical Teaching Unit and is managed by repeated escalating doses of furosemide (80 mg to over 240 mg OD in different regimens), discontinuation of the HCTZ and ramipril, and the addition of spironolactone (25 mg OD) and severe fluid restriction (750 mL per day).
- Over the next two days the patient achieves a weight loss of 2 kg (weight now 96 kg), BP is 95/50, JVP 3 cm ASA and there is less peripheral edema.
- Repeat blood work shows the following new values: serum creatinine 250 μM, serum sodium 122 mM, serum potassium 2.8 mM and serum glucose 9 mM. Urine analysis values: Urine osmolality 300 mOsm/kg, urine sodium 20 mM, and urine potassium 40 mM.

**Basic case Question 1-2: What is your assessment of the major problem?**

1. The serum sodium has dropped over the two-day period and now there is a component of acute hyponatremia
2. The low serum potassium is contributing to the drop in the serum sodium
3. **The decrease in glomerular filtration rate (GFR) is now a major contributing factor in the hyponatremia as it is decreasing the ability of the kidney to increase free water excretion ***
4. The patient has not been following the water restriction and the loss of sodium and potassium as a result of the diuretic has resulted in a worsening of the hyponatremia

**Basic case Question 1-3: To correct the abnormalities your next step in management would be to:**

1. Ensure that the water restriction is closely monitored
2. Give 3% normal saline to increase the serum sodium by 5 mM in 3 hours and then reassess
3. Order urea 30g po daily to increase free water excretion
4. **Correct the hypokalemia by giving an oral potassium supplement and check the serum magnesium ***
5. Continue the oral furosemide but change to a dose of 120 mg po three times a day (TID) and ensure the fluid restriction is in place

**Basic Case continued:**

- The patient is given potassium bicarbonate/potassium citrate (25 mEq BID for 2 days and then 25 mEq OD), the furosemide is changed to 80 mg po TID and the fluid restriction is monitored more closely.
- The spironolactone and HCTZ are continued. After two days the patient has lost a further 1.5 kg of weight, JVP is now 2-3 cm ASA, edema is less but still present, BP is 90 – 95/ 50, and pulse is 98 bpm. Chest x-ray shows resolution of the pulmonary edema but a right pleural effusion remains.
- New laboratory values: serum creatinine 230 μM, serum sodium 126 mM, serum potassium 3.5 mM, serum magnesium 0.98 mM, serum glucose 8.0 mM, urine osmolality 350 mOsm/kg, urine potassium 40 mM and urine sodium 25 mM.

**Basic case Question 1-4: Based on the progress of the previous two days, what is your plan for therapy?**

1. **Continue with the potassium supplementation, fluid restriction and switch to oral furosemide as there has been some improvement ***
2. Continue with the potassium supplementation and fluid restriction but increase the dose of diuretic to increase the weight loss
3. Start urea at a dose of 30 g po a day to increase free water loss as the response to the treatment so far has been less than optimal
4. Try to obtain tolvaptan, as the response to the treatment so far has been less than optimal

**Basic Case continued:**

- You decide to continue the same therapy. Over the next 4 days the serum sodium continues to increase to 137 mM, the serum creatinine decreases to 170 μMol/l and the weight decreases to 90 kg. The patient is able to be discharged, after being educated on watching his fluid intake.

**Basic case Question 1-5: What is the most appropriate rate of rise of serum sodium in this patient over 24 hours?**

1. **6 mEq ***
2. 2 mEq
3. 10 mEq
4. 12 mEq

**Case 2: Moderate Case**

**Case Introduction**

- A 55-year-old male is admitted in the Emergency Department for heart failure. He has a known restrictive cardiomyopathy secondary to systemic amyloidosis (amyloid light-chain) with renal involvement, and peripheral neuropathy.
- He is on chemotherapy (bortezomib and prednisone). A recent ECHO showed a non-dilated left ventricle, severe concentric hypertrophy, LVEF of 40%, tricuspid regurgitation (TR) 3/4, mitral regurgitation (MR) 2/4, pulmonary artery pressure (PAP) 55 mmHg. A coronary angiogram was normal in 2011. A transplant evaluation was performed a few months earlier and he was deemed not to be a candidate for cardiac transplant.
- He is subsequently admitted to the cardiology ward with severe fluid overload (a 15 kg weight gain since his last visit to the clinic a month earlier). On examination his weight is 90 kg, BP 100/75, heart rate (HR) 65 bpm, JVP 10 cm ASA, bilateral pulmonary crackles are present, cardiac auscultation revealed a 2/6 SEM and an S3, with massive lower limb edema.
- Laboratory values: serum sodium 132 mM, serum creatinine 250 μM, serum potassium 4.5 mM, Troponin –T High Sensitivity 25 ng/L, mildly elevated transaminases. Home medication: furosemide 80 mg BID, carvedilol 6.25 mg BID, spironolactone 25 mg OD, perindopril 2 mg OD, potassium chloride 20 mEq TID.

**Moderate case Question 2-1: Upon admission to the cardiology ward, what would be your first step in management?**

1. Increase his oral furosemide to 120mg po BID
2. **Change to intravenous (IV) furosemide, 120mg po TID ***
3. Consult Nephrology to consider starting dialysis for ultrafiltration
4. Start a vasopressin receptor antagonist (i.e. tolvaptan)

**Moderate Case continued:**

- His perindopril is held and he is initially treated with IV furosemide bolus (120 mg IV q 8h) without any response. The following day, metolazone is started with mild diuresis (700 ml over 24 hours).
- New laboratory values: serum sodium dropped to 128 mM, serum creatinine rose to 350 μM and serum potassium fell to 3.9 mM.

**Moderate case Question 2-2: What would be your next step in management?**

1. **Switch to a furosemide infusion, starting 20 mg/hr ***
2. Start hypertonic saline (3%) infusion
3. Start a vasopressin antagonist (i.e. tolvaptan)
4. Continue with the present therapy

**Moderate Case continued:**

- On the third day he is switched to a furosemide infusion with moderate diuretic response for the next two days (urine output 1.2 L per day with daily fluid restriction of 1.5 L), but his serum sodium continues to drop slowly (126 mM on the fifth day), his BP falls to 95/60 mmHg, his serum potassium falls further to 2.9 mM and his oral potassium replacement is increased to 32 mEq po of ‘slow k’ started TID.

**Moderate case Question 2-3: What would you do for treatment at this time?**

1. Consult nephrology for consideration of dialysis
2. Start IV normal saline at 100 cc/hour
3. Give hypertonic saline and diuretics together
4. Stop current therapy and start oral urea
5. **Start a vasopressin antagonist (i.e. tolvaptan) ***

**Moderate Case continued:**

- Tolvaptan (15 mg) is started on the fifth day in addition to the perfusion with marked increase in the diuresis (negative 3 L/day) and a mild improvement in serum sodium (127 mM). Serum potassium rises to 3.2 mM with supplementation.
- Tolvaptan is increased to 30 mg x 2 days with normalization of his serum sodium (136 mM) and the patient becomes euvolemic.

**Moderate Case Question 2-4: What is the most appropriate rate of rise of serum sodium in this patient over 24 hours?**

1. 2 mEq
2. 4 mEq
3. **8 mEq ***
4. Any rate of rise is fine as long as the patient is feeling okay.

**Case 3: Complex Case**

**Case Introduction:** Day 1

- A 42-year-old male with no history of cardiac disease presents to the emergency room with a four-day history of fatigue, nausea, vomiting, abdominal bloating and diarrhea following a flu-like illness.
- He is alert, but looks ill. His weight is 80 kg, BP 80/56, HR 109 bpm, temperature 38°C, respiratory rate (RR) 22 per minute and O_2_ sat 90% RA. Other than his abdomen being slightly distended, the mucus membranes are normal as is the rest of the physical exam.
- Laboratory values: serum sodium125 mM, serum creatinine 170 μM, serum potassium 3.3 mM.

**Complex Case Question 3-1: Based on your initial assessment, what would you do?**

1. Infuse hypertonic saline at 100 cc/hour for 4 hours
2. Infuse normal saline at 150 cc/hour
3. Administer urea 30g BID in a glass of water
4. Perform further tests to determine circulating volume
5. **Rapid bolus of normal saline 1 L to resuscitate BP ***

**Complex Case continued:**

- In the emergency department the patient is given 2 L of normal saline with 20 mEq/L of potassium chloride over 4 hours. Blood cultures are drawn and he is placed empirically on piperacillin/tazobactam. He is then admitted to the general medicine ward with an admission diagnosis of sepsis NYD.
- After admission to the ward he remains hypotensive and is given an additional 3 L of saline over the next 18 hours.

Day 2

- The following day, he is slightly confused, BP remains only 70/50, HR 140 bpm and his temperature is 38°C.
- Overnight his oxygen saturations start to decrease and by the morning he requires 4 L of O_2_ by nasal prongs to maintain his O_2_ saturation above 90%.

Day 3

- In the morning his condition is no better. He has an intensive care unit (ICU) consult and after assessment he is transferred to the ICU. His initial treatment in the ICU is dobutamine and norepinephrine bitartrate, which increases his BP to 105/86 but also results in an increased HR to 145 bpm and a decreased urine output to less than 30 cc/hour.
- A central line had been placed the previous day and the venous pressure is measured at >20 mmHg. Intravenous fluids are continued at 175 ml/h of normal saline with potassium chloride and Cardiology is consulted to assess the supra ventricular tachycardia.
- Laboratory values (done in the ICU): serum sodium126 mM, serum creatinine 230 μM, aspartate aminotransferase (AST) and alanine aminotransferase (ALT) grossly elevated, bilirubin 160umol/L, International normalized ratio (INR) 2.2, and lactate 5.5 mM.
- The electrocardiogram (ECG) shows sinus tachycardia and non-specific ST-T changes. The bedside ECHO shows normal size left ventricle (LV) with severe systolic function; estimated ejection fraction (EF) 15%, with 4+ MR/TR, but no thrombus or effusion**.**

**Complex Case Question 3-2: What is the most likely cause of the hyponatremia?**

1. Acute hepatitis
2. **Acute heart failure ***
3. Acute sepsis
4. Acute renal failure

**Complex Case Question 3-3: What would you do now for treatment?**

1. Infuse more saline and follow with a loop diuretic to remove free water
2. Increase to high dose urea (60 g BID)
3. Consult Nephrology to start hemodialysis
4. **Continue inotropic support with fluid restriction and loop diuretics ***

**Complex case continued:**

- The patient is transferred to the Transplant/Heart Failure service where he is given intravenous furosemide, dobutamine, milrinone, and a small dose of vasopressin IV. This results in only a minimal increase in urine output; therefore, the patient remains in an overall positive fluid balance.
- On examination his HR is 124 bpm, BP 88/56 and O_2_ saturation 90% on 50% facemask. A Swan-ganz is inserted and the readings are right atrial pressure (RAP) 22 mmHg, PAP 60/36 mmHg, pulmonary wedge pressure (PWP) 34 mmHg with V waves 60 and cardiac output of 2.3 L/min (indexed to 1.5). A cardiac biopsy is also performed.
- Repeated laboratory investigations revealed serum sodium of 123 mM, serum creatinine of 210 μM and a decrease in lactate to 2.2 mM.

Day 4 – 6

- At this point, fulminant myocarditis became the most likely diagnosis and the patient is treated with IVGG daily for 3 days. Laboratory parameters are followed and after the last dose of IVIG the serum sodium is 121mM.

**Complex Case Question 3-4: What would you do now for treatment?**

1. Intensify fluid restriction with continued diuretic use
2. **Consider the use of an antidiuretic hormone (ADH) antagonist such as tolvaptan ***
3. Begin with 3% hypertonic saline and add a diuretic
4. Continue present therapy and wait to see response in the next day or so

**Complex Case continued:**

- The decision is made to use an ADH antagonist to correct the hyponatremia and improve diuresis. Tolvaptan is started at 15 mg po daily resulting in increased urine output, avoiding the need for intubation due to fluid overload.
- There is an urgent assessment for suitability for mechanical support. Repeated electrolytes in 6 hours reveal the serum sodium has increased to 124 mM, serum creatinine is 190 μM, while the increased urine output is resulting in a net negative fluid balance.
- The patient remains stable until the next morning when the heart rate begins to increase and there is a rise in the serum lactate level to 4.5mM. Urgent arrangements are made for temporary Impella insertion into LV via percutaneous cut down in left axilla.
- After this procedure the patient has a change in medications to milrinone only, post operation. A second dose of tolvaptan (15 mg) and IV furosemide are given 1 hour prior to surgery. After the successful Impella insertion his hemodynamics improve: BP 100/70, HR 109 bpm, temperature 37°C and saturated O_2_ is 96% on 30% FiO_2_, still intubated. The hemodynamic parameters also show improvement: RAP 12 mmHg, PAP 44/20 mmHg and PWP 22 mmHg. The repeat serum sodium was 129 mM, serum creatinine 165 μM and lactate 1.2 mM.

Day 7

- The following day the patient is extubated, urine output continues to be good and the hemodynamic profile is good. The laboratory investigations reveal that the serum sodium has increased to 130 mM, the serum creatinine is 150 μM and the serum lactate level is normal.
- The cardiac biopsy shows acute lymphocytic myocarditis, with no eosinophilia, giant cells, or Langerhans cells. All cultures are negative; therefore, the antibiotics are stopped. A repeat ECHO shows improvement in LVEF to 25%.
- Another dose of tolvaptan (15 mg po) is given and IV furosemide is continued with good result. The elevated liver enzymes and function tests take another week to return to normal levels.

**Complex Case Question 3-5: What is the most appropriate rate of rise of serum sodium in this patient over 24 hours?**

1. **6 mEq ***
2. 2 mEq
3. 10 mEq
4. Any rate of rise as this has been more acute than chronic

**Complex Case continued:**

Day 8

- The following day, the serum sodium is 135mM, serum creatinine is 145 μM and the patient is in a negative fluid balance of 2 L. A repeat ECHO shows the LVEF unchanged at 25% and the Impella is working well. Tolvaptan is not given but IV furosemide is continued and an angiotensin-converting-enzyme (ACE) inhibitor and beta-blocker therapy is initiated at starting doses.

 Day 9- 20

- The following day, the serum sodium is 138 mM despite being an additional 2 L negative in total fluid balance. There is continued improvement in the overall condition and by the twelfth day (five days after initial insertion), LV function has increased to an EF of 45% and the Impella is successfully removed via local anesthesia.
- Over the course of the next eight days the patient is ambulated, tolerates the ACE inhibitor and beta-blocker therapies and is able to be discharged on the ACE inhibitor, beta-blocker and 20mg of furosemide po OD. The serum sodium on discharge is 139 mM and the LVEF 50%.

**Complex Case Question 3-6: The serum sodium was normal at hospital discharge, why?**

1. The tolvaptan cured the problem
2. The patient is now euvolemic, which necessitates normal serum sodium
3. The patient was no longer receiving excessive free water
4. **The initial cause for hyponatremia, low effective circulating volume, was no longer present ***
