# Supplementary figures and images for: Approach to hyponatremia in congestive heart failure: a survey of Canadian specialist physicians and trainees
Source: Can J Kidney Health Dis. 2016 Jan 20;3:4. doi: 10.1186/s40697-016-0094-9 (PMC4719575; doi:10.1186/s40697-016-0094-9)

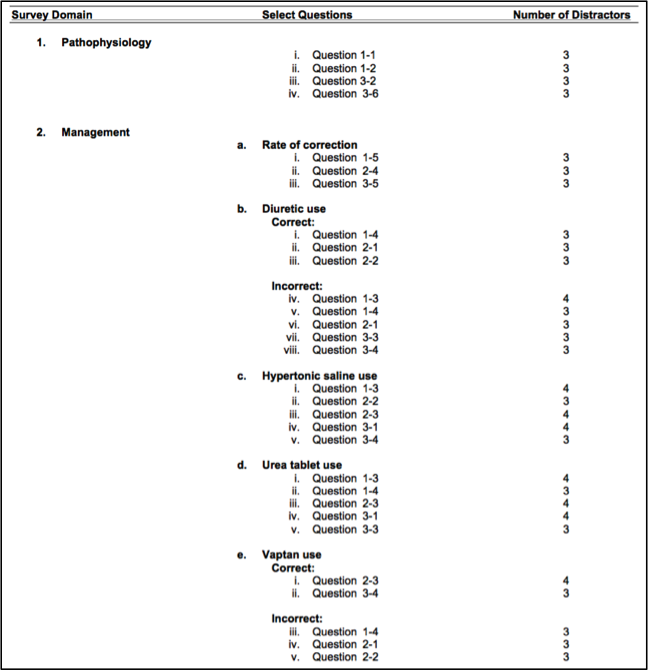

Supplement: Additional file 2: — Individual questions assessing the pathophysiology and management strategies for hyponatremia. The complete list of questions included in the assessment of the pathophysiology and varying management strategies for hyponatremia are listed. The number of incorrect responses (distractors) for each multiple-choice question are included for completeness. (PNG 126 kb) [file 40697_2016_94_MOESM2_ESM.png]
